# Impact of Extracellular pH on Apoptotic and Non-Apoptotic TRAIL-Induced Signaling in Pancreatic Ductal Adenocarcinoma Cells

**DOI:** 10.3389/fcell.2022.768579

**Published:** 2022-02-24

**Authors:** Sofie Hagelund, Anna Trauzold

**Affiliations:** Institute for Experimental Cancer Research, University of Kiel, Kiel, Germany

**Keywords:** TRAIL, TRAIL receptor, apoptosis, non-apoptotic signaling, extracellular pH, pancreatic ductal adenocarcinoma

## Abstract

Tumor necrosis factor-related apoptosis-inducing ligand (TRAIL) is an important mediator of tumor immune surveillance. In addition, its potential to kill cancer cells without harming healthy cells led to the development of TRAIL receptor agonists, which however did not show the desired effects in clinical trials. This is caused mainly by apoptosis resistance mechanisms operating in primary cancer cells. Meanwhile, it has been realized that in addition to cell death, TRAIL also induces non-apoptotic pro-inflammatory pathways that may enhance tumor malignancy. Due to its late detection and resistance to current therapeutic options, pancreatic ductal adenocarcinoma (PDAC) is still one of the deadliest types of cancer worldwide. A dysregulated pH microenvironment contributes to PDAC development, in which the cancer cells become highly dependent on to maintain their metabolism. The impact of extracellular pH (pH_e_) on TRAIL-induced signaling in PDAC cells is poorly understood so far. To close this gap, we analyzed the effects of acidic and alkaline pH_e_, both in short-term and long-term settings, on apoptotic and non-apoptotic TRAIL-induced signaling. We found that acidic and alkaline pH_e_ differentially impact TRAIL-induced responses, and in addition, the duration of the pH_e_ exposition also represents an important parameter. Thus, adaptation to acidic pH_e_ increases TRAIL sensitivity in two different PDAC cell lines, Colo357 and Panc1, one already TRAIL-sensitive and the other TRAIL-resistant, respectively. However, the latter became highly TRAIL-sensitive only by concomitant inhibition of Bcl-xL. None of these effects was observed under other pH_e_ conditions studied. Both TRAIL-induced non-apoptotic signaling pathways, as well as constitutively expressed anti-apoptotic proteins, were regulated by acidic pH_e_. Whereas the non-apoptotic pathways were differently affected in Colo357 than in Panc1 cells, the impact on the anti-apoptotic protein levels was similar in both cell lines. In Panc1 cells, adaptation to either acidic or alkaline pH_e_ blocked the activation of the most of TRAIL-induced non-apoptotic pathways. Interestingly, under these conditions, significant downregulation of the plasma membrane levels of TRAIL-R1 and TRAIL-R2 was observed. Summing up, extracellular pH influences PDAC cells’ response to TRAIL with acidic pH_e_ adaptation, showing the ability to strongly increase TRAIL sensitivity and in addition to inhibit TRAIL-induced pro-inflammatory signaling.

## Introduction

Pancreatic cancer is currently the seventh leading cause of cancer deaths worldwide, despite being relatively infrequent (Bray et al., 2018; Rawla et al., 2019; Christopher et al., 2020). The 5-year survival rate remains low (9%–10%), due to poor early detection, inadequate therapy options, and no available screening tests (Christopher et al., 2020; American-Cancer-Society, 2021). Pancreatic ductal adenocarcinoma (PDAC) accounts for more than 90% of all pancreatic cancer cases and occurs in the exocrine pancreas (Christopher et al., 2020; American-Cancer-Society, 2021). Both acini and ductal cells, comprising the exocrine pancreas, have a structured network of ion channels that enables them to regulate pH in the lumen and interstitium, as well as an intracellular pH (pH_i_) (Novak et al., 2013; Chii et al., 2014; Pallagi et al., 2015). Thus, pancreatic epithelial cells form a unique and dynamic pH microenvironment (Pedersen, Novak, et al., 2017), and dysregulation of this particular microenvironment can contribute to PDAC development (Chii et al., 2014; Pedersen et al., 2017; Schnipper et al., 2020). Here, the cancer cells become highly dependent on the altered pH regulation to maintain their metabolism (Swietach 2019). The majority of cancers have a dysregulated extracellular pH (pH_e_) microenvironment due to a change in glucose metabolism known as the “Warburg effect,” where they produce high amounts of lactate and protons even in the presence of oxygen (Vander Heiden et al., 2009; Kato et al., 2018). The accumulation of lactate and protons contributes to tumor acidosis through ion channels, while at the same time, pH_i_ is maintained (Chii et al., 2014). Ultimately, this favors tumor progression and therapy resistance (Chii et al., 2014; Vaupel et al., 2019).

Though pH_i_ is generally well-maintained at a neutral state, it responds to changes in pH_e_, meaning that pH_e_ can influence cellular physiology through pH_i_ regulation achieved by signaling proteins sensitive to changes in pH_e_ (Boron, 2004; Michl et al., 2019; Swietach, 2019). Cells exposed to acidic stress will generally not demonstrate ideal pH_i_ homeostasis, and steady-state pH_i_ drops when (even well-perfused single) cells are exposed to acidic pH_e_ (Boron, 2004; Kato et al., 2013; Swietach, 2019). This could be the consequence of cancer cells trying to protect themselves from lactate retention, which would alternatively take place if the pH_i_ was considerably higher than pH_e_ (Swietach, 2019). Moreover is it well-known that intracellular acidification (a decrease of 0.3–0.4 pH_i_ values) occurs in mammalian cells during apoptosis (Matsuyama et al., 2000; Lagadic-Gossmann et al., 2004; Sergeeva et al., 2017) and has been observed after multiple types of apoptotic stimuli such as UV exposure, growth factor deprivation, death receptor-mediated and mitochondria-mediated apoptosis (Lagadic-Gossmann et al., 2004). To that extent, intracellular acidification has been shown to be either caspase-independent or caspase-dependent (Lagadic-Gossmann et al., 2004). Due to this knowledge, pH_e_ could affect the apoptotic response.

Tumor necrosis factor-related apoptosis-inducing ligand (TRAIL), a member of the TNF superfamily (TNFSF), can bind to four different plasma membrane receptors in humans: TRAIL-receptors 1, 2, 3, and 4 (TRAIL-R1, 2, 3, and 4). While TRAIL-R1 and TRAIL-R2 can induce apoptosis through their death domains, TRAIL-R3 and TRAIL-R4 are lacking this ability since they miss or have a truncated death domain, respectively. Although the function of TRAIL-R3 and TRAIL-R4 is still not well understood, it is likely that they can work as decoys and regulatory receptors (von Karstedt et al., 2017). Following the binding of TRAIL, the formation of the death-inducing signaling complex (DISC) is initiated. Within the DISC, the adapter protein FADD is recruited, which in turn leads to recruitment and activation of caspases-8 and/or -10 (Dickens et al., 2012). In so-called type I cells, sufficient levels of activated caspase-8/10 are generated at the DISC for direct activation of the effector caspases required for the activation of the caspase cascade. In contrast, type II cells require the engagement of the mitochondrial apoptosis pathway for the efficient activation of caspases (Ozören and El-Deiry, 2002; Jost et al., 2009). In these cells, activation of small amounts of caspase-8 in DISC leads to the cleavage of Bid, which in turn leads to Bax/Bak-mediated mitochondrial outer membrane permeabilization (MOMP) (Luo et al., 1998; Antonsson et al., 2001; Dewson et al., 2009; Huang et al., 2016). Upon MOMP, the second mitochondrial activator of caspases/direct inhibitor of apoptosis-binding protein with a low isoelectric point (pI) (SMAC/DIABLO) is released to the cytosol for counteracting protein X-linked inhibitor of apoptosis protein (XIAP) (Ozören and El-Deiry, 2002; Jost et al., 2009), an inhibitor of effector caspases (Deveraux et al., 1998; Bratton et al., 2001; Holcik and Korneluk, 2001). In addition, cytochrome c is released, the step which is a prerequisite for the formation of the apoptosome. In apoptosome, another initiator caspase, caspase-9, is activated, which in turn is able to fully activate the effector caspase-3 to trigger apoptosis in type II cells (Riedl and Salvesen, 2007; Kalkavan and Green, 2018). Of note, PDAC cells have been shown to employ a type II apoptotic signaling pathway upon death receptor stimulation (Hinz et al., 2000). The observations that TRAIL death receptors are frequently overexpressed in cancer and that TRAIL induces apoptosis in tumor cells and not normal healthy cells led to the development of TRAIL/TRAIL-R-based therapeutic drugs and their testing in clinical trials (Lemke et al., 2014; de Miguel et al., 2016). However, despite promising pre-clinical findings, none of these drugs showed the desired effects in cancer patients. These disappointing results can be attributed to the apoptosis resistance mechanisms present in many primary tumor cells and may comprise the constitutive upregulation of the anti-apoptotic proteins operating at different levels of the TRAIL/TRAIL-R signaling cascades like decoy/regulatory receptors, FLIP, Bcl-xL, and IAP (Hinz et al., 2000; Trauzold et al., 2001; Trauzold et al., 2003; Lemke et al., 2010). It also becomes obvious that cancer cells frequently misuse TRAIL death receptors as an inducer of pro-inflammatory pathways, like NF-κB, ERK1/2, Akt, Src, p38, and JNK. These non-canonical TRAIL-induced pathways become visible in apoptosis-resistant tumors and by enhancing cell proliferation, migration, and invasion may lead to cancer progression (Trauzold et al., 2001; Ehrenschwender et al., 2010; von Karstedt et al., 2015; Azijli et al., 2013; Trauzold et al., 2006; Hoogwater et al., 2010).

Importantly, the TRAIL/TRAIL-R system represents one of the mechanisms by which the immune system contributes to the surveillance of developing tumors (Falschlehner et al., 2009). In addition, cancer cells themselves frequently express TRAIL. Consequently, in the (patho)physiological context, the importance of TRAIL/TRAIL-R signaling cannot be underestimated. Since PDAC cells originate from cells naturally exposed to acidic and alkaline pH and an acidic environment is observed often in tumors, it is very likely that pH_e_ influences the response to TRAIL in these cells. Yet, such data are not available so far. Therefore, in the present study, we sought to investigate the impact of both the short-term and long-term exposition to alkaline (7.6) and acidic (6.5) pH_e_ compared to control (pH_e_ 7.4) on TRAIL-induced apoptotic and non-apoptotic signaling in PDAC cells.

## Materials and Methods

### Cell Culture and Reagents

Human pancreatic ductal adenocarcinoma cell lines Panc1 and Colo357 were cultured in RPMI-1640 medium (Sigma-Aldrich, Hamburg, Germany) supplemented with 10% FCS (Pan BioTech, Aidenbach, Germany), 10 mM d-glucose (Sigma-Aldrich), 5% GlutaMax (Gibco, Darmstadt, Germany), and 5% sodium pyruvate (Gibco) under standard cell culture conditions (37°C, 5% CO_2_) up to 70%–85% confluence. The pH medium 7.6 and 6.5 were obtained by using the Henderson–Hasselbalch equation [for further information, we refer to Michl et al. (2019)] and done similarly to Czaplinska et al. (2020), Yao et al. (2020), and Flinck et al. (2018) by adjusting the HCO_3_ concentration by adding a proper amount of NaHCO_3_ (Sigma-Aldrich) and NaCl to ensure equal osmolarity. Panc1 pH_e_ (7.6 and 6.5)-adapted cell lines were established and kindly provided by Stine Pedersen (University of Copenhagen, Denmark). Colo357 pH_e_ (7.6 and 6.5)-adapted cell lines were generated internally. Human sTRAIL/Apo2L (Peprotech, Hamburg, Germany) was used to treat (200 ng/ml) PDAC cells. Trypsin (Pan BioTech) was used to detach cells when passaged, while Accutase (Sigma-Aldrich) was used to detach cells before experiments. PBS (Gibco) was used during washing steps. Cell lines were authenticated by STR profiling and tested regularly for *Mycoplasma* contamination.

### Experimental pH_e_ Setup

Cells were either exposed to an “acute” pH_e_ setup, with a different pH_e_ and exposure times between 24 h and a maximum of 7 days depending on the experiment, or they were cultured for a period of minimum 30 days in a certain pH_e_ value and stocked for future use as pH_e_ “adapted” cells.

### Colony Formation Assay

For each cell line and each pH_e_ condition, 1 × 10^3^ cells/well were seeded in six-well plates. Twenty-four hours later, cells were treated with TRAIL (200 ng/ml) for another 24 h, and after removing the medium and replacing it with a fresh one, they were allowed to grow for additional 5–7 days. The colonies were quantified by NyOne (Synentec, Elmshorn, Germany) live-cell imager. Finally, the cells were stained with 0.5% crystal violet (Sigma-Aldrich)/20% MeOH (ROTH, Karlsruhe, Germany) to visualize the colonies. Crystal violet was thoroughly washed away with ddH_2_O, and plates were left to dry.

### Live-Cell Imaging Staining

The cells (1.5 × 10^4^ per well) were seeded in 96-well plates for 24 h, followed by TRAIL treatment (200 ng/ml) for another 24 h. The inhibitors Navitoclax (Selleck Chemicals, Distributor Absource Diagnostics GmbH, München, Germany) and Venetoclax (Selleck Chemicals) were added in a concentration of 5 µM to the cells 2 h prior to TRAIL (200 ng/ml) treatment. Hoechst “Bisbenzimid H 33342” (2.5 µg/ml, Sigma-Aldrich), Calcein-AM (1 µg/ml, BioLegend, San Diego, United States), and propidium iodide (PI) (Invitrogen, Karlsruhe, Germany) (10 µg/ml) mixed in PBS were added to the wells and incubated for 20 min in the cell incubator (37°C, 5% CO_2_). Pictures were obtained and quantified by NyOne (Synentec). Quantification of both, dead cells and live cells, at the same time was used to clarify whether the decreased number of viable cells results from enhanced cell death or decreased cell proliferation.

### Flow Cytometry

Flow cytometry was performed on either BD FACSCalibur™ (Becton Dickinson, Heidelberg, Germany) or MACSQuantify™ (Miltenyi Biotec, Bergisch Gladbach, Germany) and evaluated with CellQuest Pro (Becton Dickinson) or MACSQuantify™ software (Miltenyi Biotec), respectively.

### Cell Surface TRAIL Receptor Expression

Cell surface expression levels of TRAIL receptors were analyzed by flow cytometry. Briefly, 4.5 × 10^5^ cells were seeded per well in six-well plates for 24 h. Then, cells were detached from culture dishes by treatment with Accutase (Sigma-Aldrich), re-suspended in 0.05% NaN_3_/PBS, transferred to a plate with V-shaped wells (Nerbe plus, Winsen, Germany), and centrifuged at 300 × *g* for 10 min at 4°C. The supernatant was discarded, and cells were incubated for 30 min with the following APC-conjugated antibodies with the concentration 1:3 in 0.6% BSA/PBS: TRAIL-R1 (FAB347A), TRAIL-R2 (FAB6311A), TRAIL-R3 (FAB6302A), and TRAIL-R4 (FAB633A) all purchased from R&D Systems GmbH, Wiesbaden, Germany. Respective isotype control stainings were performed with APC-conjugated mouse IgG1 Control (IC002A) and mouse IgG2B (IC0041A) (both from R&D Systems GmbH). Finally, cells were washed in 0.05% NaN3/PBS, re-suspended in 1% PFA (Morphisto, Offenbach am Main, Germany)/PBS, and the staining was measured within 24 h by flow cytometry. A population size of 10,000 cells was regarded as representative for data evaluation.

### Crystal Violet Assay

Cells (1.5 × 10^4^ per well) were seeded in 96-well plates for 24 h, followed by TRAIL treatment (200 ng/ml) for another 24 h. The supernatant was discarded, and adherent cells were incubated with crystal violet as already described in the *Colony Formation Assay* section. After wells were dried, they were filled with 200 µl of 100% MeOH and incubated for 20 min at room temperature with gentle shaking. Finally, absorption was measured on either Tecan Sunrise or Tecan Infinite M200 Pro with wavelength at 590 nm and reference at 700 nm.

### Western Blotting

Cells were lysed in RIPA buffer supplemented with Complete Protease Inhibitor Cocktail and PhosphoStop (both from Roche, Mannheim, Germany), and western blot analyses were performed as described previously (Trauzold et al., 2003). Primary antibodies were purchased from the following: Cell Signaling, Frankfurt, Germany [anti-ERK1/2 (9102), anti-phospho-ERK1/2 (9106), anti-JNK (9252), anti-phospho-JNK (9255), anti-p38 (9212), anti-phospho-p38 (9216), anti-Akt (2920), ant-phospho-Akt (4058), anti-IκBα (4814), anti-phospho-IκBα (2859), anti-Src (2110), anti-phospho-Src (2101), anti-TRAIL-R2 (3696), anti-caspase-8 (9746), anti-caspase-3 (9668), anti-Survivin (2802), anti-XIAP (2045), anti-cIAP2 (3130), and anti-Mcl-1 (94296)]; Santa Cruz Biotechnology, Heidelberg, Germany [anti-cIAP1 (sc-7943) and anti-HSP90 (sc-7947)]; BD Pharmingen, Heidelberg, Germany [anti-Bcl-x (516446)]; Merck Millipore, Darmstadt, Germany [anti-TRAIL-R1 (AB16955)]; Enzo Life Sciences, Lörrach, Germany [anti-FLIP (ALX-804961)]; and from Sigma-Aldrich [anti-β-actin (A5441)]. Bound primary antibodies were detected by using HRP-linked secondary antibodies [Cell Signaling, anti-mouse IgG (7076) and anti-rabbit IgG (7074)]. Membranes were developed using Pierce™ ECL (Thermo Fisher), Pierce™ ECL plus (Thermo Fisher), Radiance Chemiluminescence Subtrat (Azure), Radiance Q (Azure), or Radiance Plus (Azure) and the machines AGFA curix50 (with CL-X Posure Film from Thermo Fisher; developer and fixer from AGFA) or Azure Imaging Systems 300Q. Densitometric analyses were carried out using ImageJ software (Schneider et al., 2012).

### Statistics

Data are presented as mean ± S.E.M of at least three independent experiments unless otherwise mentioned. Data analyses were performed using GraphPad Prism 7.0. When several groups were analyzed, one-way or two-way ANOVA with either Tukey’s or Sidak’s multiple comparison tests was used, which was dependent on whether pH conditions or treatment conditions were compared, respectively. *p*-values of <0.05 were considered statistically significant; *p < 0.05, **p < 0.01, ***p < 0.001, and ****p < 0.0001.

## Results

### Acidic pH_e_ Increases TRAIL-Induced Cell Death in PDAC Cells

**FIGURE 1:**
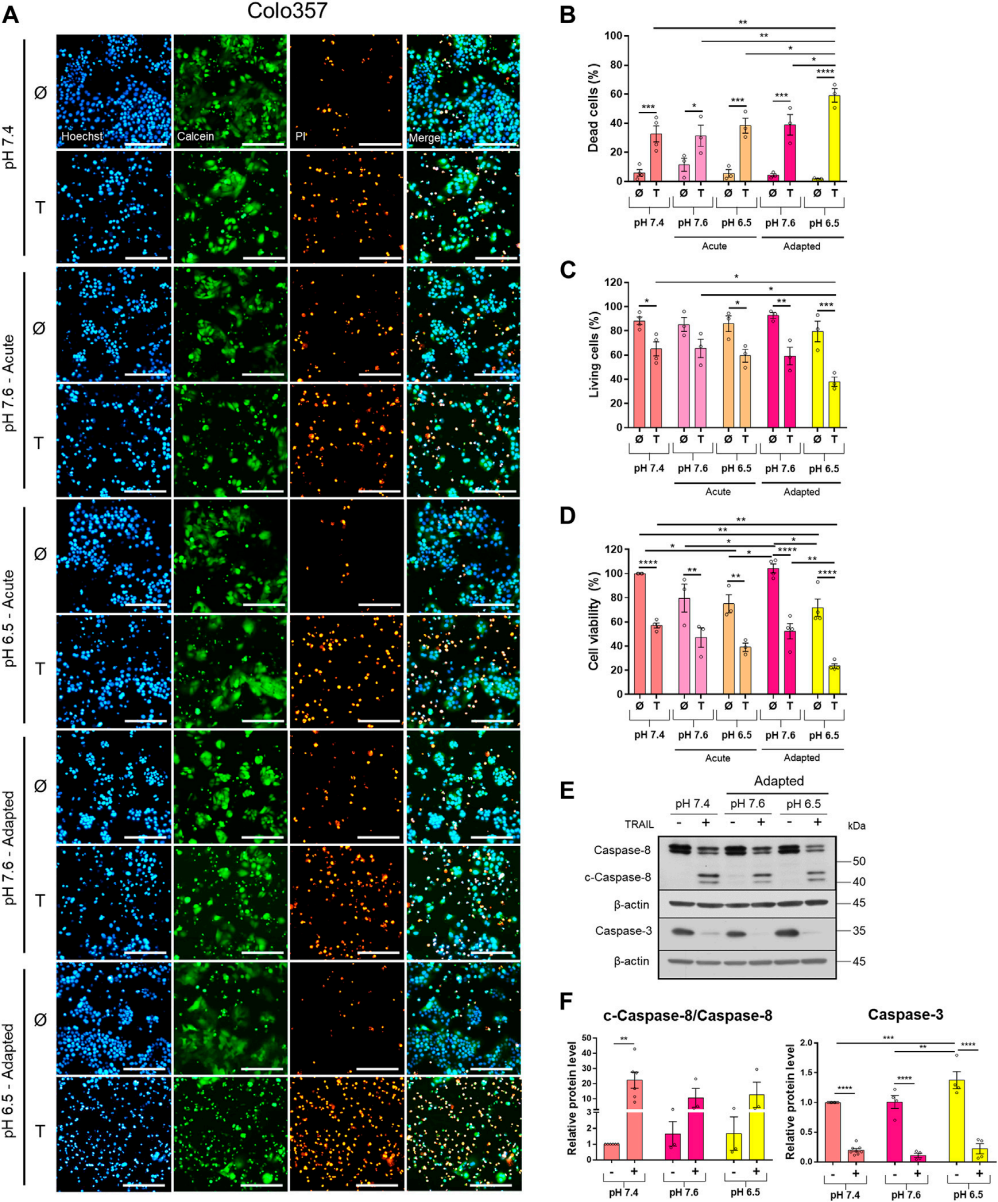
Acidic pH_e_ increases TRAIL-induced cell death in the TRAIL-sensitive cell line Colo357. Twenty-four hours after seeding, cells were treated for additional 24 h with TRAIL. Cell death/viability was determined by live-cell staining. **(A)** Colo357 cells were live-stained with Hoechst, Calcein-AM, and propidium iodide (PI), and all measurements were processed by NyOne. Scale bar = 200 µm. **(B)** Quantification of PI-positive dead cells. **(C)** Quantification of Calcein-positive living cells. Hoechst was used to stain the nuclei. **(D)** Cell viability measured by crystal violet staining. Data are normalized to untreated cells cultured under pH_e_ 7.4 conditions and presented as cell viability in percentage without (Ø) or with 200 ng/ml TRAIL (T) for 24 h. Data are shown as mean with S.E.M error bars, of at least three independent experiments per cell line. *p < 0.05, ** < 0.01, *** < 0.001, **** < 0.0001: Significant difference between untreated and treated using two-way ANOVA with Sidak’s multiple-comparisons test or between pH_e_ conditions using two-way ANOVA with Tukey’s multiple comparisons test. **(E)** Colo357 cells were grown for 24 h, then either not treated (−) or treated (+) with 200 ng/ml TRAIL for 24 h, lysed, and subjected to western blot analyses for caspase-8 (c-caspase-8 = cleaved-caspase-8) and caspase-3. Blots are representatives of at least three independent experiments. **(F)** Densitometric quantification normalized to loading control and the respective level of untreated cells cultured under pH_e_ 7.4 conditions. Data are shown as mean with S.E.M error bars, of at least three independent experiments per cell line. ** < 0.01, *** < 0.001, and **** < 0.0001: significant difference of the results was calculated between untreated and treated samples using two-way ANOVA with Sidak’s multiple-comparison test or between pH_e_ conditions using two-way ANOVA with Tukey’s multiple-comparison test.

**FIGURE 2:**
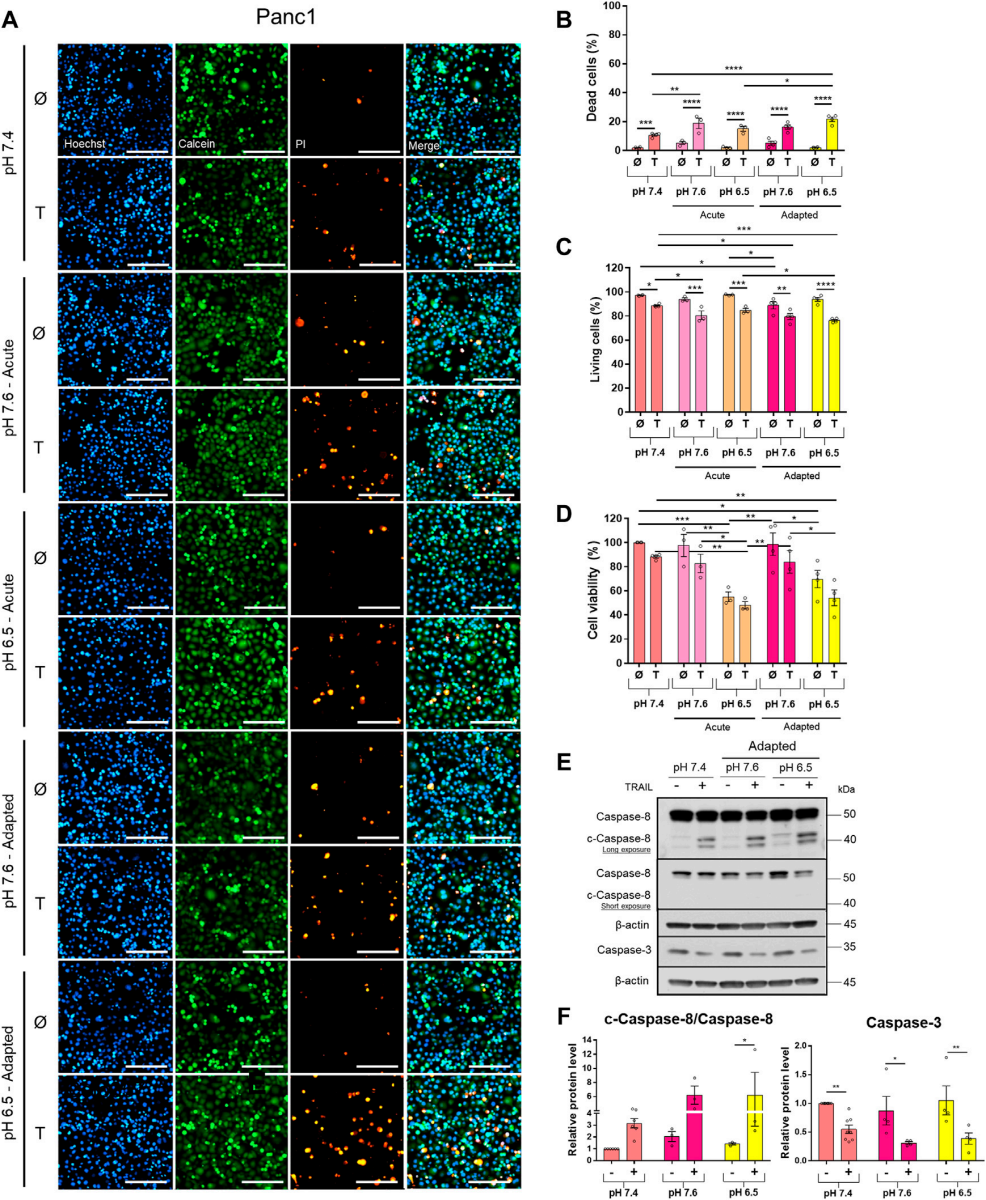
Acidic pH_e_ increases TRAIL-induced cell death only slightly in the TRAIL-resistant cell line Panc1. Twenty-four hours after seeding, cells were treated for additional 24 h with TRAIL. Cell death/viability was determined by live-cell staining. **(A)** Panc1 cells were live-stained with Hoechst, Calcein-AM, and propidium iodide (PI), and all measurements were processed by NyOne. Scale bar = 200 µm. **(B)** Quantification of PI-positive dead cells. **(C)** Quantification of Calcein-positive living cells. Hoechst was used to stain the nuclei. **(D)** Cell viability measured by crystal violet staining. Data are normalized to untreated cells cultured under pH_e_ 7.4 conditions and presented as cell viability in percentage without (Ø) or with 200 ng/ml TRAIL (T) for 24 h. Data are shown as mean with S.E.M error bars, of at least three independent experiments per cell line. *p < 0.05, ** < 0.01, *** < 0.001, **** < 0.0001: significant difference between untreated and treated using two-way ANOVA with Sidak’s multiple-comparison test or between pH_e_ conditions using two-way ANOVA with Tukey’s multiple-comparison test. **(E)** Panc1 cells were grown for 24 h, then either not treated (−) or treated (+) with 200 ng/ml TRAIL for 24 h, lysed, and subjected to western blot analyses for caspase-8 (c-caspase-8 = cleaved-caspase-8) and caspase-3. Blots are representatives of at least three independent experiments. **(F)** Densitometric quantification normalized to loading control and the respective level of untreated cells cultured under pH_e_ 7.4 conditions. Data are shown as mean with S.E.M error bars of at least three independent experiments per cell line. *p < 0.05 and **<0.01: significant difference of the results was calculated between untreated and treated samples using two-way ANOVA with Sidak’s multiple comparisons test or between pH_e_ conditions using two-way ANOVA with Tukey’s multiple-comparison test.

To study the impact of pH_e_ on TRAIL-induced signaling in PDAC cells, we established Colo357 and Panc1 cell lines adapted for a longer period of time to either of the three pH_e_ conditions: pH_e_ 6.5 (simulating pH_e_ in the tumor microenvironment), pH_e_ 7.6 (corresponding to the luminal pH_e_ in the exocrine pancreas), or pH_e_ 7.4 (control). Following adaptation, cells were exposed to TRAIL for 24 h, and cell viability and cell death were studied by live-cell staining with Hoechst, Calcein-AM, and PI, followed by quantification of the living and dead cells by the NyOne live-cell imager. In agreement with previous data (Hinz, Trauzold et al., 2000; Trauzold et al., 2003), Colo357 cells were re-proven to be sensitive (Figure 1) while Panc1 cells to be resistant to TRAIL-mediated cell death (Figure 2). Interestingly, acidic pH_e_ adaptation strongly sensitized Colo357 cells to TRAIL treatment, decreasing cell viability (Figure 1C; pH_e_ 7.4: 65.1%, pH_e_ 6.5 adapted: 38.0%) and correspondingly increasing cell death (Figures 1A,B; pH_e_ 7.4: 32.7%, pH_e_ 6.5 adapted: 59.3%). In contrast, under these conditions, only a slight enhancement of TRAIL-induced cell death was detectable in Panc1 cells (Figures 2A,B; pH_e_ 7.4: 10.8%, pH_e_ 6.5 adapted: 21.7%). No significant difference in TRAIL sensitivity could be detected between cells adapted to pH_e_ 7.6 and 7.4 (Figures 1, 2). In addition, we investigated the impact of acutely changed pH_e_ on TRAIL-mediated cell death. For this purpose, we exposed cells to different pH_e_ shortly before (24 h) and during the treatment with TRAIL. Short-term exposition of cells to pH_e_ of either 7.6 or 6.5 did not have an impact on TRAIL sensitivity neither in Colo357 (Figure 1) nor Panc1 cells with the only exception of acute pH 7.6 exposure, which still exhibited lower TRAIL sensitivity than cells adapted to acidic pH_e_ (Figure 2). Similar results for both pH_e_ settings (adapted and acute) have been obtained using crystal violet staining as an indicator of cell viability (Figures 1D, 2D).

Western blot analyses of apoptotic caspases revealed that in Colo357 pH_e_-adapted cells, TRAIL induced strong caspase-8 and caspase-3 activation, irrespective of the culture conditions (Figure 1E). In agreement with the results of cell death-quantifying assays (Figure 1B), acidic pH_e_ adaptation potentiated TRAIL-induced cleavage of both caspases in these cells (Figures 1E,F). Again, the effects observed in TRAIL-treated Panc1 cells were less pronounced than those in Colo357 cells (Figures 2E,F).

**FIGURE 3:**
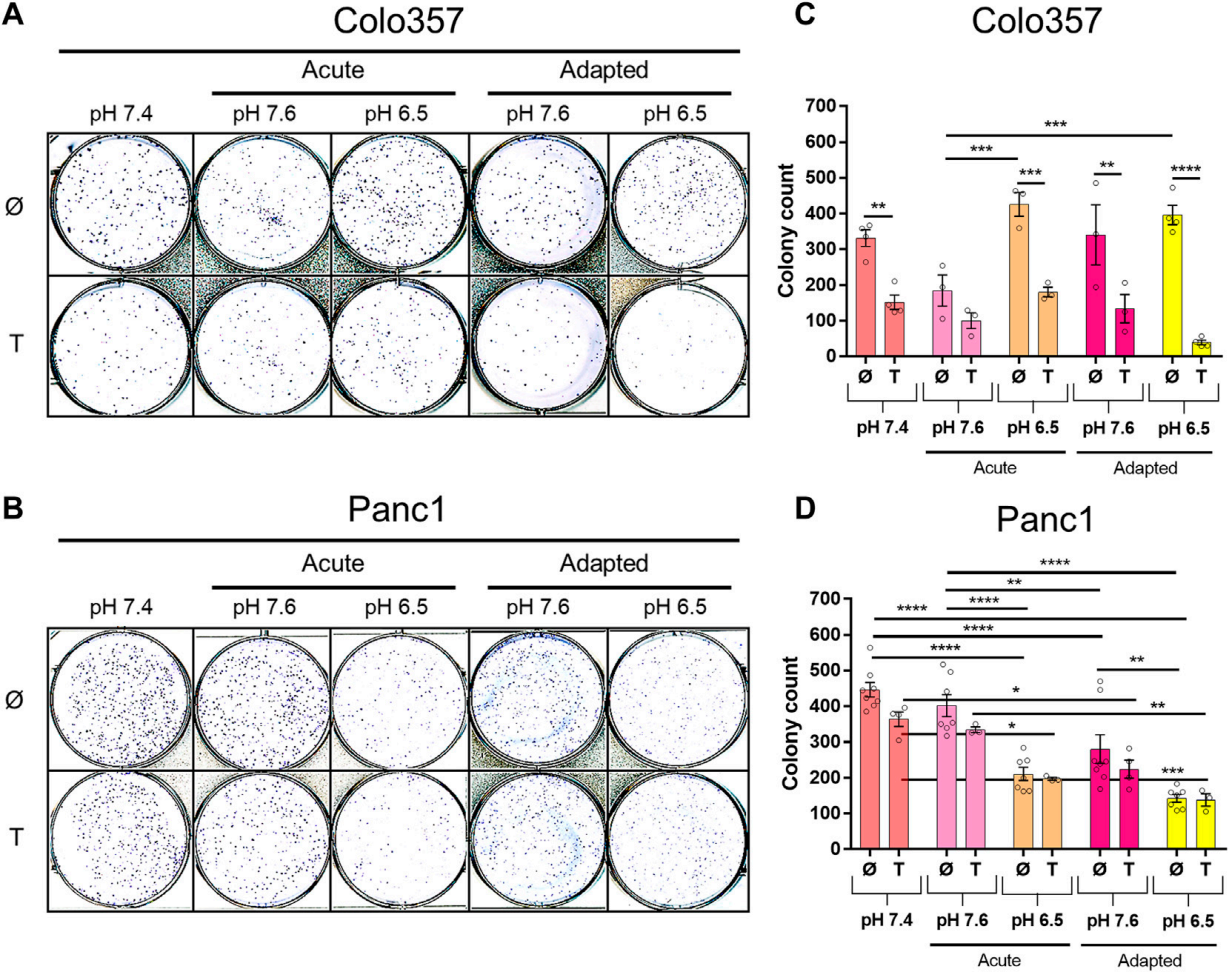
pH_e_ and TRAIL affect colony formation in PDAC cells. Cells (1 × 10^3^ per well) were seeded to six-well plates for 24 h, treated with TRAIL for 24 h, and following medium change let to grow for additional 5–7 [Colo357; **(A,C)**] or 5–6 days [Panc1, **(B,D)**]. The colonies were counted by live-cell imager NyOne. **(A,B)** Representative crystal violet-stained plates and **(C,D)** quantified data shown as mean with S.E.M error bars of at least three independent experiments per cell line. *p < 0.05, ** <0.01, *** <0.001, **** < 0.0001: Significant difference between untreated and treated cells using two-way ANOVA with Sidak’s multiple-comparison test or between pH_e_ conditions using two-way ANOVA with Tukey’s multiple-comparison test.

Next, we studied the clonogenic survival of both PDAC cell lines under different pH_e_ conditions with and without TRAIL treatment. As shown in Figure 3, in this aspect, Colo357 cells (Figures 3A,C) and Panc1 cells (Figures 3B,D) respond differently to pH_e_. Thus, whereas acute and long-term adaptation to acidic pH_e_ as well as adaptation to pH_e_ of 7.6 resulted in a robustly reduced clonogenic survival of Panc1 cells, none of these conditions did affect Colo357 cells. In Colo357 cells, solely the acute exposition to pH_e_ 7.6 decreased the number of colonies, an effect which was not observed in Panc1 cells. Consistent with previous data (Legler, Hauser et al., 2018), treatment with TRAIL for 24 h significantly reduced clonogenic survival of Colo357 cells cultured under normal pH_e_ conditions of 7.4. In contrast, TRAIL did not affect clonogenic survival in Panc1 cells, when each pH_e_ condition was compared to their respective pH_e_ control.

### Acidic pH_e_ Affects Non-Apoptotic TRAIL-R Signaling in PDAC Cells

**FIGURE 4:**
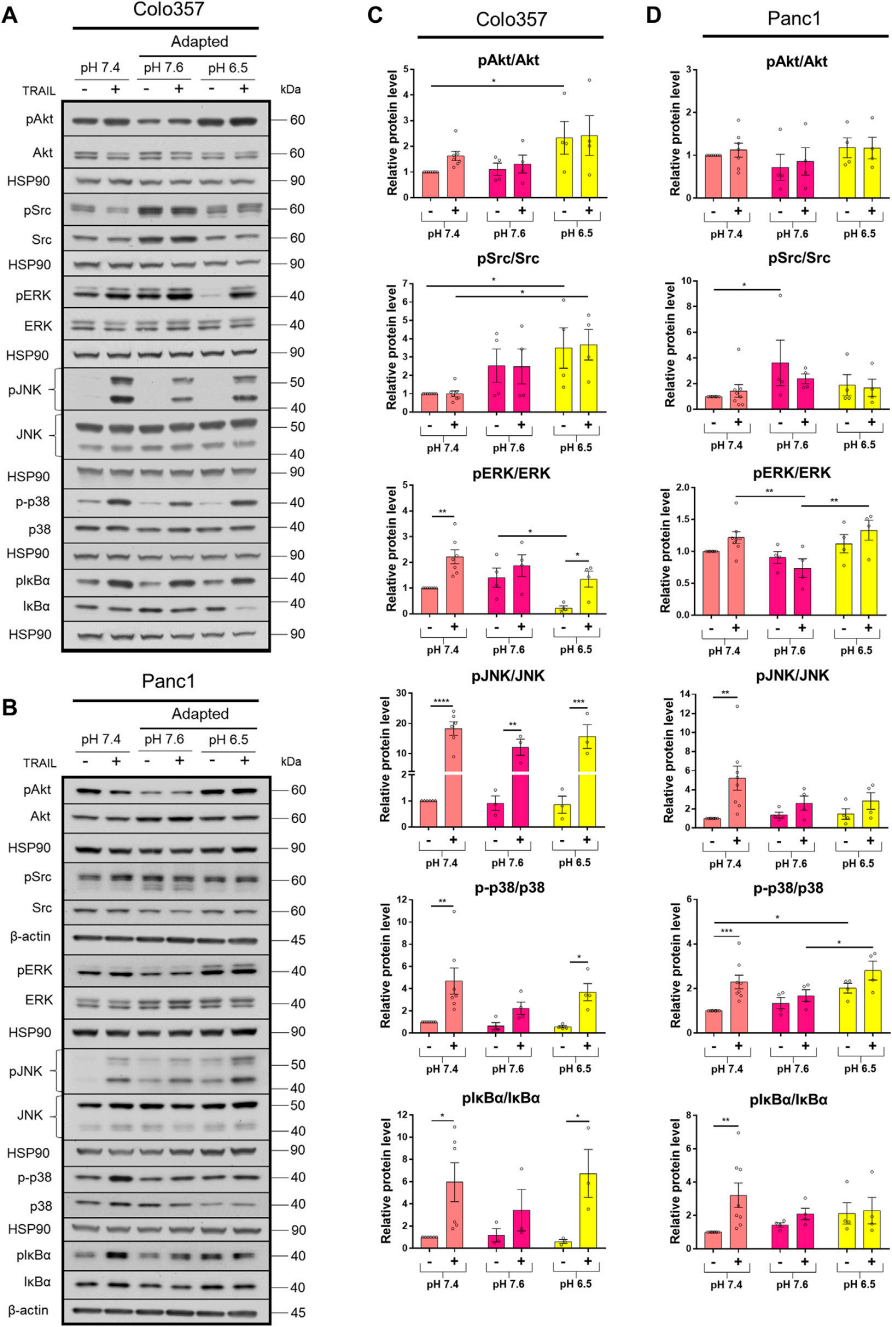
Acidic pH_e_ affects non-apoptotic TRAIL-R signaling in PDAC cells. **(A,B)** PDAC cells were grown for 24 h, then either not treated (−) or treated (+) with 200 ng/ml TRAIL for 3 h, lysed, and subjected to western blot analyses for pAkt, Akt, pSrc, Src, pERK, ERK, pJNK, JNK, p-p38, p38, pIκBα, and IκBα. Blots are representative and show **(C,D)** densitometric quantification normalized to loading control and the respective level of untreated cells cultured under pH_e_ 7.4 conditions. Phosphorylated (p) proteins were normalized to non-phosphorylated proteins. Data are shown as mean with S.E.M error bars, of at least three independent experiments per cell line. *p < 0.05, **<0.01, ***<0.001, and ****<0.0001: significant difference between untreated and treated cells using two-way ANOVA with Sidak’s multiple-comparison test or between pH_e_ conditions using two-way ANOVA with Tukey’s multiple-comparison test.

The TRAIL-induced apoptotic pathway is frequently inhibited in cancer cells leading to the de-masking of the potential of TRAIL death receptors to induce several pro-inflammatory signaling pathways, which may ultimately result in tumor progression (von Karstedt et al., 2017; Trauzold et al., 2001; Trauzold et al., 2006; Hoogwater et al., 2010; Azijli et al., 2010). To study the impact of pH_e_ on the activation of these non-canonical TRAIL-induced pathways, cells grown under different pH_e_ conditions were exposed to TRAIL for 3 h. Subsequently, the phosphorylation/activity of Akt, Src, and MAP kinases, as well as the phosphorylation of the Iκbα as an indicator for the activation of NFκB, was analyzed by western blot using phospho-specific antibodies. As a control, the overall cellular expression levels of these proteins were analyzed in parallel. Under normal pH_e_ conditions, TRAIL treatment resulted, in both PDAC cell lines, in strong activation-related phosphorylation of p38, JNK, and IκBα (Figure 4; Supplementary Figure S1). In addition, in Colo357 cells, TRAIL also led to the activation of ERK1/2 in pH_e_ 7.4 and especially in acidic pH_e_ compared to their respective pH_e_ controls (Figure 4A, C; Supplementary Figures S1A,C). Neither in Colo357 cells nor Panc1 cells, changes in Src or Akt activity could be observed following exposure to TRAIL (Figure 4; Supplementary Figure S1). Of note, these proteins became significantly more active in Colo357 cells adapted to acidic pH_e_ compared to control cells or cells adapted to pH_e_ of 7.6 (Figures 4A,C). No such effects were seen in Panc1 cells (Figures 4B,D). Regarding the impact of pH_e_ on TRAIL-induced non-canonical pathways, the adaptation of Colo357 cells to acidic pH_e_ did not change their response to TRAIL (Figures 4A,C). Also, no significant TRAIL-mediated phosphorylation of ERK1/2, p38, or IκBα was observed in these cells when adapted to pH_e_ of 7.6 (Figures 4A,C). Importantly, the adaptation of Panc1 cells to either pH_e_ 7.6 or 6.5 strongly reduced their ability to activate the non-canonical signal transduction pathways in response to TRAIL (Figures 4B,D). The activity of the non-canonical TRAIL-induced signaling pathways was also investigated in acute pH_e_ exposure (Supplementary Figure S1). In general, a stronger response to TRAIL treatment was observed in Panc1 cells compared to their pH_e_-adapted counterparts (Supplementary Figures S1B,D), whereas more similar response patterns, at least for the acidic pH_e_ were detected in Colo357 cells (Supplementary Figures S1A,C). In these cells, alkaline acute pH_e_ exposure resulted in higher non-apoptotic activity compared to alkaline adapted Colo357 cells (Supplementary Figures S1A,C).

### pH_e_ Influences the Expression of TRAIL Death Receptors

**FIGURE 5:**
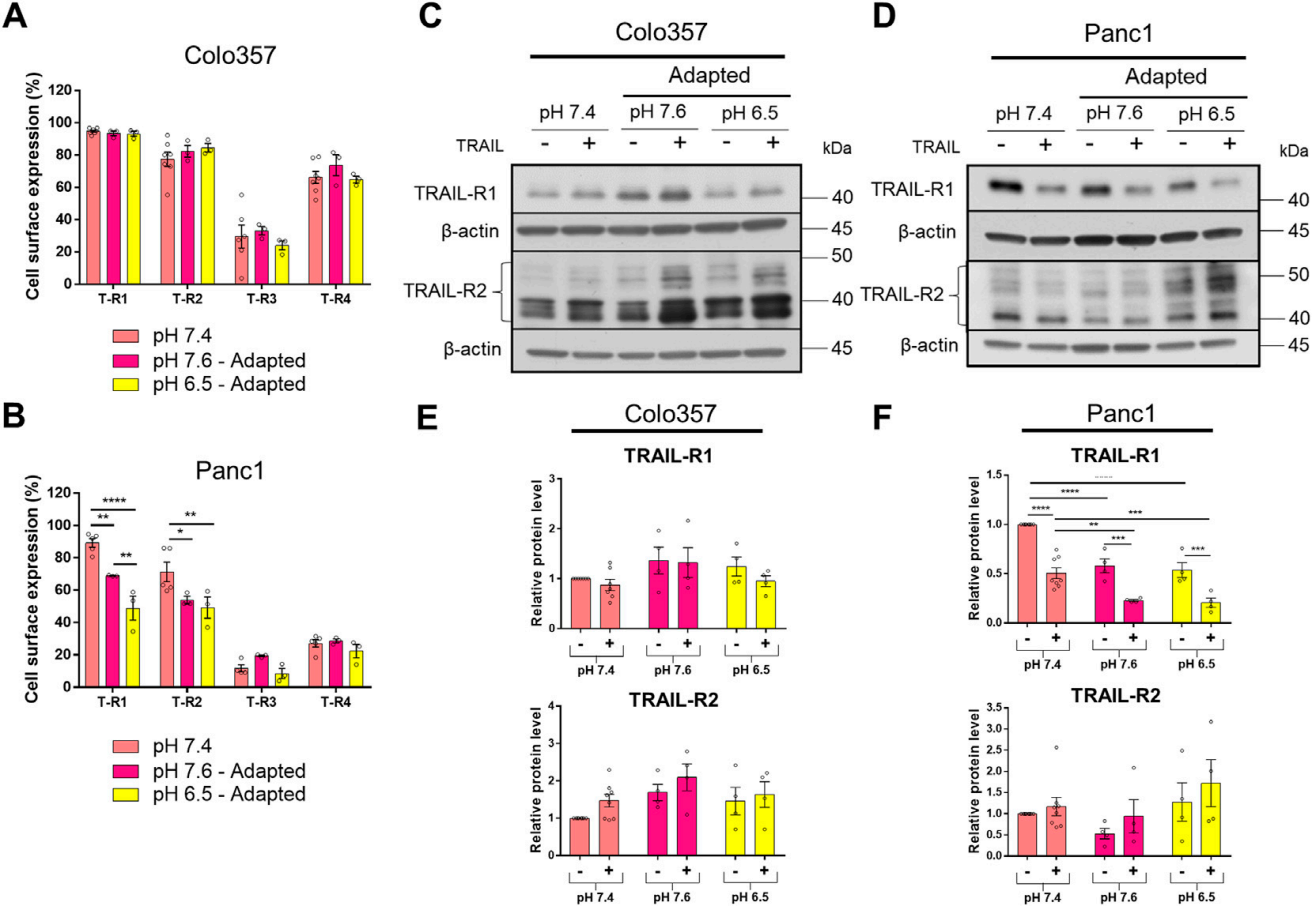
pH_e_ influences the expression of TRAIL death receptors. **(A,B)** PDAC cells were grown for 24 h, then stained with APC-conjugated anti-TRAIL-Rs antibodies to measure their cell surface expression by flow cytometry. **(C,D)** PDAC cells were grown for 24 h, then either not treated (−) or treated (+) with 200 ng/ml TRAIL for 24 h, lysed, and subjected to western blot analyses for TRAIL-R1 and TRAIL-R2. Blots are representative and **(E,F)** show densitometric quantification normalized to loading control and the respective level of untreated cells cultured under pH_e_ 7.4 conditions. Data are shown as mean with S.E.M error bars, of at least three independent experiments per cell line. **<0.01, ***<0.001, and ****<0.0001: significant difference between untreated and treated cells using two-way ANOVA with Sidak’s multiple-comparison test or between pH_e_ conditions using two-way ANOVA with Tukey’s multiple-comparison test.

The data presented so far show that PDAC cells adapted to acidic pH_e_ became more sensitive to TRAIL compared to those cultured at pH_e_ 7.4 or 7.6. Yet, this holds true non-restrictive only for Colo357 cells, while Panc1 cells were only marginally affected and stayed largely TRAIL-resistant. To gain insights into the potential mechanisms behind these effects, we next investigated the expression of TRAIL-Rs as well as several anti-apoptotic proteins in cells adapted to different pH_e_ conditions and treated or not with recombinant TRAIL for 24 h. Flow cytometric analyses revealed no impact of pH_e_ on the cell surface expression of TRAIL death receptors in Colo357 cells (Figure 5A). Likewise, no differences in the cell surface expression of TRAIL-R3 or TRAIL-R4 were detected neither in Colo357 cells nor in Panc1 cells (Figures 5A,B).

Intriguingly, in Panc1 cells, both TRAIL-R1 and TRAIL-R2 were significantly decreased at the plasma membrane following their adaptation to either pH_e_ 7.6 or 6.5 (Figure 5B). Of note, TRAIL-R1 appeared to be stronger influenced by pH_e_ than TRAIL-R2. In addition, acidic adaptation reduced the plasma membrane levels of TRAIL-R1 significantly stronger than adaptation to pH_e_ of 7.6 (Figure 5B).

Western blot analyses of whole-cell lysates were mostly congruent with the results obtained for the cell surface-expressed TRAIL-R1 and TRAIL-R2. Thus, no pH_e_-dependent changes in the levels of these receptors were observed in Colo357 cells (Figures 5C,E) while cellular levels of TRAIL-R1 in Panc1 cells were significantly reduced in cells adapted to both pH_e_ 7.6 and 6.5 (Figures 5D,F). However, and contrary to the data showed for the cell surface expression, no impact of pH_e_ on cellular levels of TRAIL-R2 could be detected by western blot (Figures 5D,F).

Besides differences in the impact of pH_e_ on TRAIL-Rs levels, treatment with TRAIL also differentially influenced these receptors in Colo357 cells and Panc1 cells. Concretely, TRAIL had no effects on the overall levels of TRAIL-R1 or TRAIL-R2 in Colo357 cells in none of the studied pH_e_ conditions (Figures 5C,E). In contrast, in Panc1 cells treatment with TRAIL led to a strong reduction of TRAIL-R1 levels, but interestingly did not affect the levels of TRAIL-R2 (Figures 5D,F).

The expression of TRAIL-Rs was also investigated following the acute pH_e_ exposure (Supplementary Figure S2) and delivered almost completely contrary results. Thus, instead of being downregulated, the levels of TRAIL-R1 were increased in Panc1 cells acutely exposed to either 7.6 or 6.5 (Supplementary Figures S2B,D). Similar effects were observed in Colo357 cells in which in acidic pH_e_ additionally TRAIL-R2 levels were significantly upregulated (Supplementary Figures S2A,C). Moreover, upon TRAIL-treatment TRAIL-R1 levels decreased not only in Panc1 cells but also in Colo357 cells, an effect which was not seen in pH_e_ adapted Colo357 cells (Supplementary Figure S2). However, whereas in Panc1 cells this effect was observed under all pH_e_ conditions, TRAIL-induced downregulation of TRAIL-R1 could be detected only under acidic pH_e_ (Supplementary Figures S2A,C).

### pH_e_ Affects the Expression Levels of Anti-Apoptotic Proteins

**FIGURE 6:**
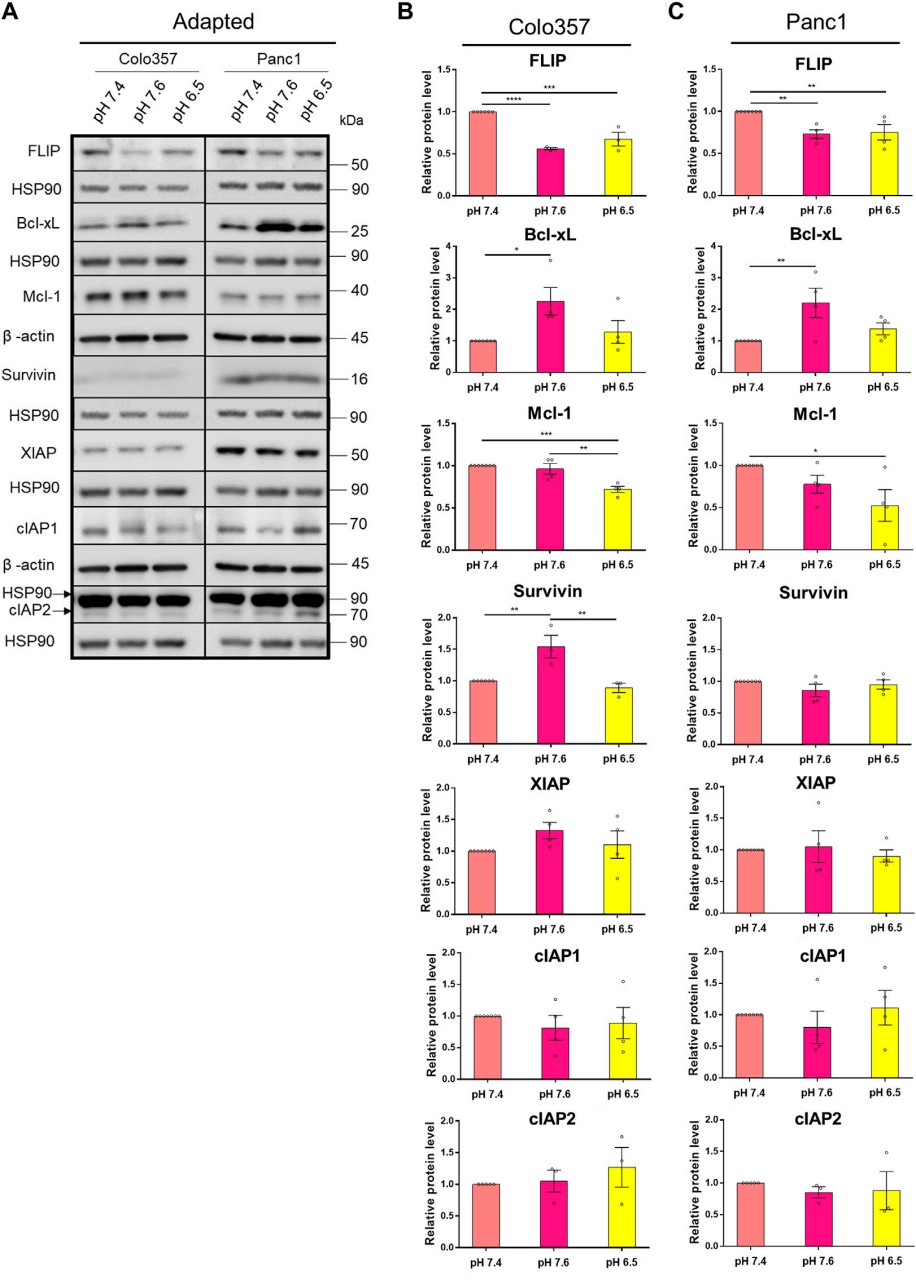
pH_e_ affects the expression levels of anti-apoptotic proteins. **(A)** PDAC cells were grown for 27 h, then lysed and subjected to western blot analyses for FLIP, Bcl-xL, Mcl-1, Survivin, XIAP, cIAP1, and cIAP2. Blots are representative, where Colo357 cells and Panc1 cells were compared side by side. **(B,C)** Densitometric quantification normalized to loading control and the respective level of untreated cells cultured under pH_e_ 7.4 conditions. Data are shown as mean with S.E.M error bars, of at least three independent experiments per cell line. *p < 0.05, **<0.01, ***<0.001, and ****<0.0001: significant difference between pH_e_ conditions using one-way ANOVA with Tukey’s multiple-comparison test.

Apoptosis resistance due to an overexpression of anti-apoptotic proteins is a known hallmark of cancer (Hanahan and Weinberg, 2011). Concerted upregulation of proteins operating at different steps of the apoptotic signal transduction pathway was shown to assure TRAIL resistance in PDAC cells (Hinz et al., 2000; Trauzold et al., 2003). However, to the best of our knowledge, the effects of pH_e_ on the expression levels of these proteins in PDAC cells are unknown so far. To close this gap, we next analyzed by western blot the levels of FLIP, Mcl-1, Bcl-xL, and the members of the inhibitor of apoptosis (IAP) family Survivin, XIAP, cIAP1, and cIAP2 in whole-cell lysates of Colo357 and Panc1 cells cultured in different pH_e_ conditions. As shown in Figure 6, in both cell lines, albeit stronger in Colo357 than in Panc1 cells, FLIP was significantly decreased in both pH_e_ 7.6 and 6.5 adapted cell lines compared to control (Figures 6A–C). In both pH_e_ 6.5 adapted cell lines, Mcl-1 was also significantly decreased compared to control, and again stronger in Colo357 cells (Figures 6A–C). Interestingly, Bcl-xL significantly increased in both pH_e_ 7.6 adapted cells lines compared to control (Figures 6A–C). Regarding the expression levels of IAPs, only Survivin and only in Colo357 cells showed pH_e_-dependence being significantly increased in pH_e_ 7.6 adapted cells (Figures 6A,B; Supplementary Figure S3). None of the remaining analyzed IAP proteins (XIAP, cIAP1, and cIAP2) show any significant changes due to changes in pH_e_ either in Colo357 or in Panc1 cells (Figures 6A–C). In agreement with already published data (Hinz, Trauzold, et al., 2000; Trauzold, Schmiedel, et al., 2003), and with the apoptosis-resistant phenotype of Panc1 cells, these cells showed higher expression of most of the analyzed anti-apoptotic proteins (FLIP, Bcl-xL, Survivin, and XIAP) as compared to Colo357 cells except for Mcl-1, which was higher in Colo357 cells (Figure 6A).

The expression of the anti-apoptotic proteins has been also studied in cells acutely exposed to different pH_e_ (Supplementary Figure S4). Interestingly, the expression pattern was not the same as for the cells adapted to pH_e_. Thus, the levels of the studied proteins in cells acutely exposed to different pH_e_ were either the same or even slightly increased compared to control (Supplementary Figure S4). These results show again that cells react differently to long-time and acute pH_e_ exposure.

### Inhibition of Bcl-xL Highly Sensitizes Acidic pH_e_-Adapted Panc1 Cells to TRAIL-Induced Cell Death

**FIGURE 7:**
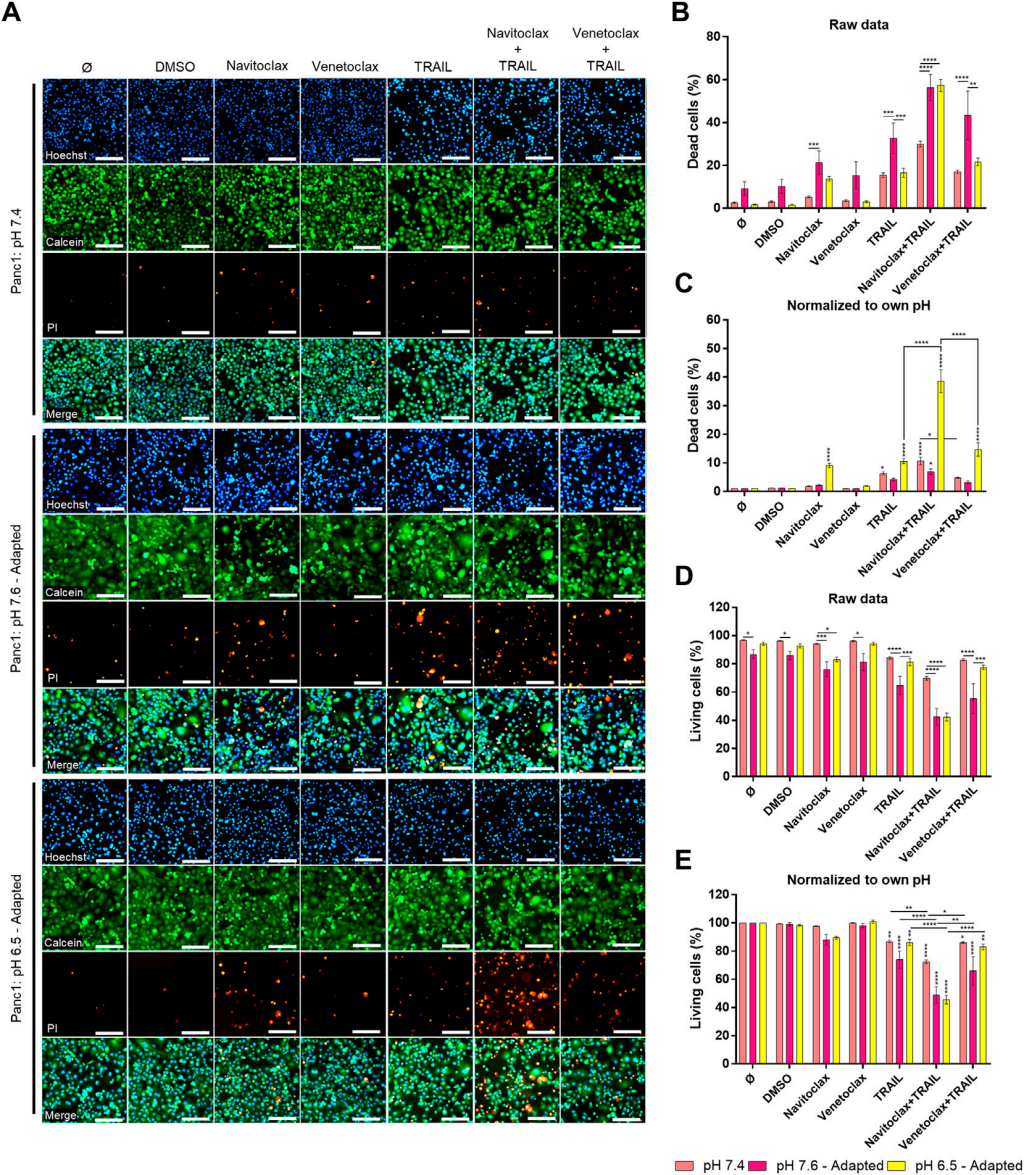
Navitoclax (Bcl-xL inhibitor) sensitizes Panc1 cells to TRAIL-induced cell death in acidic pH_e_. **(A)** Panc1 cells were grown for 24 h, then either not treated (Ø) or treated with DMSO, 5 µM inhibitor (Navitoclax or Venetoclax) for 26 h, TRAIL treatment (200 ng/ml) for 24 h, or a combination of pre-treated 5 µM inhibitor for 2 h prior to TRAIL treatment for an additional 24 h. Cells were finally live-stained with Hoechst, Calcein-AM, and propidium iodide (PI), and all measurements were processed by NyOne. Scale bar = 200 µm. **(B,C)** Cell death quantification of PI-positive cells, either showed as raw data or normalized to respective pH_e_ control. **(D,E)** Living cells quantified from Calcein-positive cells, either showed as raw data or normalized to respective pH_e_ control. Hoechst was used to stain the nuclei. Data are shown as mean with S.E.M error bars, of at least three independent experiments per cell line. *p < 0.05, **<0.01, ***<0.001, and ****<0.0001: significant difference between untreated and treated cells using two-way ANOVA with Sidak’s multiple-comparison test or between pH_e_ conditions using two-way ANOVA with Tukey’s multiple-comparison test. Significant differences in “raw data” are only shown between different pH conditions within each treatment. Significant differences in “normalized to own pH” are only shown between different treatments within the same pH, where vertical stars are significant differences to respective controls (Ø or DMSO).

Our results revealed that under acidic pH_e_, Colo357 cells become highly sensitive to TRAIL, while this effect was only marginal in Panc1 cells. Since the latter overexpress Bcl-xL, we wonder whether inhibition of Bcl-xL could sensitize these cells to TRAIL under acidic pH_e_ conditions. Recently, the so-called BH3-mimetics have been developed, which bind to and neutralize the activity of anti-apoptotic members of the Bcl-2-family. Among several generated and pre-clinically evaluated BH3-mimetics, Navitoclax (ABT-263) and Venetoclax (ABT-199) have successfully entered clinical trial testing. Navitoclax potently antagonizes Bcl-2 and Bcl-xL, whereas Venetoclax selectively inhibits Bcl-2. We set out to investigate whether BH3-mimetics may harbor the potential to sensitize Panc1 cells to TRAIL and to study their potentially synergistic effects with acidic pH_e_. Panc1 cells adapted to different pH_e_ were pre-treated for 2 h with inhibitors prior to treatment with TRAIL for an additional 24 h. Cell viability and cell death were analyzed by staining the cells with Hoechst, Calcein-AM, and PI followed by measurement and quantification on the live-cell imager NyOne. As shown in Figures 7A–C, under normal pH_e_ conditions, Navitoclax only marginally increased TRAIL-mediated cell death. Likewise, it did not show synergizing effects with TRAIL in cells adapted to pH_e_ 7.6 (Figures 7A–C). Intriguingly, Navitoclax robustly enhanced the death-inducing capacity of TRAIL in Panc1 cells adapted to acidic pH_e_. In contrast, pre-treatment with Venetoclax did not enhance TRAIL-induced apoptosis further, highlighting the pivotal role of Bcl-xL in mediating apoptosis resistance in Panc1 cells (Figures 7A–C). The same patterns were obtained when analyzing cell viability using cell staining with Calcein-AM (Figures 7A,D,E), except that a combination of Navitoclax with TRAIL was also able to significantly decrease the cell viability in alkaline pH_e_-adapted cells compared to TRAIL treatment only (Figures 7A,D,E). Again, no decrease in cell viability could be observed when combining TRAIL with Venetoclax compared to TRAIL alone (Figures 7A,E). Similar results were generated by studying cell viability using crystal violet staining (Supplementary Figure S5).

Summing up, we provide here a comprehensive analysis of the effects of pH_e_ on TRAIL-induced apoptotic and non-apoptotic signaling in PDAC cells. We show that these pathways are differentially affected by alkaline and acidic pH_e_. Most importantly, we found that cells adapted to acidic pH_e_ become highly sensitive to TRAIL-induced cell death either when treated with TRAIL as a single agent or in combination with the already clinically approved drug Navitoclax (ABT-263).

## Discussion

Dysregulated pH_e_ microenvironment, commonly observed in PDAC, influences many physiological and cellular processes in tumor cells (Kato et al., 2018). pH_e_ in tumors is generally more acidic than in normal tissue and can decrease to as low as 5.8 (Tannock and Rotin, 1989). In our study, we included both cells acutely exposed and adapted to either alkaline or acidic pH_e_. We consider selecting and adapting cells to a specific pH_e_ to be a highly relevant biological setup, in order to determine how cancer pH_e_ microenvironment influences cell signaling. Additionally, we compared both pH_e_ setups, as we speculated that this would be affecting PDAC cells differently. To our knowledge, most studies have focused exclusively on acute pH_e_ exposure and acidic pH_e_, and the latter is presumably because acidic pH_e_ has been observed in many tumors (Tannock and Rotin, 1989). In our study, we included alkaline pH_e_ as well, because this is particularly interesting concerning PDAC due to the fact that the pancreas normally produces a high amount of bicarbonate into the lumen (Pallagi et al., 2015).

\We found that when PDAC cells were either exposed acutely or adapted to acidic pH_e_, the colony formation decreased in Panc1 cells but was unaffected in Colo357 cells. For the alkaline pH_e_ setups, there were differences between acute and adapted pH_e_ conditions for both cell lines. In Panc1 cells, colony formation remained unchanged in acute alkaline pH_e_ exposure but decreased when adapted and was the complete opposite for Colo357 cells. No studies concerning the influence of pH_e_ on colony formation in PDAC have been conducted according to our knowledge, but in equivalence to our study, one study showed that Panc1 cells, but not Colo357 cells, decreased in growth significantly after acute pH_e_ 6.5 exposure (Kumar et al., 2010). Similar to Panc1 cells, lung carcinoma decreased in growth under acute acidic pH_e_ exposure (Sutoo et al., 2020). Another study showed that very short exposure (10–30 min) of alkaline pH_e_ decreased cell area in breast cancer (Khajah et al., 2013), similar to Colo357 cells (data not shown).

Acidic pH_e_ has been shown to be able to potentiate TRAIL-induced cell death in some cancer types such as prostate carcinoma (Lee et al., 2004), colorectal carcinoma (Lee et al., 2004), gastric carcinoma (Hong and Han 2018), colon carcinoma (Meurette et al., 2007), hepatocarcinoma (Meurette et al., 2007), and lung cancer (Valiahdi et al., 2013). According to our knowledge, corresponding studies have not yet been performed with PDAC cells. Likewise, no data on the impact of alkaline pH_e_ on TRAIL-induced signaling is available so far. Therefore, in the present study, we addressed this issue and compared side by side the TRAIL-induced death and pro-inflammatory signaling in TRAIL-sensitive and TRAIL-resistant PDAC cell lines Colo357 and Panc1 cells, respectively. We observed increased cell death particularly in acid-adapted Colo357 cells, but only slightly in Panc1 cells, which stayed highly resistant even in acidic pH_e_. In agreement, increased activity of pro-apoptotic proteins was observed in acid-adapted Colo357 cells, but to a much lower degree in acid-adapted Panc1 cells, correlating well with cell death observations in both cell lines. Interestingly, both cell lines became more vulnerable when grown in alkaline pH_e_, where a higher number of dead cells were observed even without treatment with TRAIL. Cancer cells need to maintain an optimal pH_i_ value, which is often kept slightly more alkaline or the same as in healthy cells (Chii et al., 2014). A sudden change in pH_e_ can disrupt pH_i_ (Boron, 2004; Kato et al., 2013; Michl et al., 2019; Swietach 2019), which could last either shortly or for a longer period, usually reflected by the cells' ability to regulate metabolism, ion channels, and metabolite transporters (Vander Heiden et al., 2009; Chii et al., 2014; Vaupel et al., 2019). Thus, growing cells in alkaline pH_e_ may disrupt important cellular processes making them more vulnerable. Especially, PDAC is known to be highly dependent on autophagy, a nutrient-scavenging process (Yang et al., 2011; Flinck et al., 2020), where lysosomes, a compartment with a pH_i_ as low as 4.7 for optimal hydrolytic enzyme activity, play an important role in this process (Casey et al., 2010; Halcrow et al., 2021).

TRAIL sensitivity can be altered at many different cellular levels. The protein expression of TRAIL-Rs has already been seen to be regulated in gastric cancer cells acutely exposed to acidic pH_e_, where both TRAIL-R1 and TRAIL-R2 increased (Hong and Han, 2018), similar to our results with acutely exposed Panc1 cells. Interestingly, adaptation to acidic pH_e_ had the opposite effect on TRAIL-R1 in Panc1 cells. To this extent, both TRAIL-R1 and TRAIL-R2 significantly decreased at the cell surface in acid-adapted Panc1 cells. Thus, it is worth noting that Panc1 cells exhibit lower cell surface expression of TRAIL-R2 than TRAIL-R1 and that they have a preference for TRAIL-R2 compared to TRAIL-R1 for the induction of apoptosis (Nahacka et al., 2018). In our study, the protein level and cell surface expression of TRAIL-R1 in Panc1 cells correlated, suggesting that it was not a change in cellular location but rather a lower expression of TRAIL-R1. Alternatively, enhanced constitutive receptor internalization and subsequent degradation could account for the lower levels of TRAIL-R1 in pH_e_-adapted cells. Specific degradation of TRAIL-R1 has already been described as an effect of steady-state receptor internalization and modification by the membrane-associated RING-CH ubiquitin ligase (March-8) (van de Kooij, et al., 2013). Ultimately, this outcome would contribute to TRAIL resistance. Ligand-induced endocytosis and subsequent degradation of TRAIL-R1 could also account for the observed lower levels of this receptor in Panc1 cells following TRAIL treatment. Interestingly, we also found that TRAIL-R2 decreased at the cell surface in both acidic and alkaline pH_e_-adapted Panc1 cells, and while this correlated well with the total cellular protein level in alkaline-adapted Panc1 cells (mean decrease of ∼50%), it did not in the acid-adapted Panc1 cells. This suggests that TRAIL-R2 showed changed intracellular distribution in acid-adapted Panc1 cells, an effect that has already been demonstrated to occur to a high degree in Panc1 cells under normal pH_e_ conditions (Haselmann et al., 2014). Summing up, the relocation of TRAIL-R1 and TRAIL-R2 may be one of the mechanisms Panc1 cells utilize to maintain TRAIL resistance. Furthermore, we clearly show that the exposure time to pH_e_ (acute or adapted) influences the overall and the plasma membrane level of TRAIL-Rs.

In cancer cells, TRAIL death receptors regularly induce a non-canonical signaling pathway, which becomes of high relevance especially in apoptosis-resistant cells (Trauzold et al., 2001; Ehrenschwender et al., 2010; von Karstedt et al., 2015; Azijli et al., 2013; Trauzold et al., 2006; Hoogwater et al., 2010). Here, we observed a generally higher activity of these pathways in Colo357 cells upon TRAIL treatment compared to Panc1 cells. In particular, in Panc1 cells adapted to either alkaline or acidic pH_e_, almost no differences in the TRAIL-induced activation of IκBα, p38, and JNK were observed. This could be due to the much lower cell surface expression of both TRAIL-R1 and TRAIL-R2 in pH_e_-adapted Panc1 cells, which ultimately would cause a general lower activation of all pathways these two receptors may induce upon triggering. Because TRAIL can initiate both the pro-apoptotic and non-apoptotic pathways, it may not be surprising to see the non-apoptotic proteins increase significantly more in acid-adapted Colo357 cells. Alkaline-adapted Colo357 cells showed mostly decreased or the same activity upon TRAIL treatment compared to control or acid-adapted Colo357 cells. Similar tendencies were seen in breast cancer cells where acute alkaline pH_e_ conditions reduced the levels of activated p38, Akt, and ERK1/2 (Khajah et al., 2013). Even though acidic pH_e_ increases TRAIL-induced cell death, it is worth noting that the non-apoptotic pathway might be activated to a higher degree as well. Hence, if the apoptotic pathway is not superior, TRAIL treatment can induce and select for malignant progression.

Anti-apoptotic proteins can be responsible for decreased cell death while keeping non-apoptotic signaling ongoing, which contributes to malignant aggressiveness (Hinz et al., 2000; Trauzold et al., 2001; Trauzold et al., 2003; Lemke et al., 2010). In our study, we showed that some of these proteins are influenced by pH_e_. TRAIL-induced cytochrome c release has been seen to increase in acute acidic pH_e_ compared to normal pH_e_ in prostate carcinoma, while multiple anti-apoptotic proteins (cFLIP, cIAP1, cIAP2, and Bcl-2) remained overall unchanged (Lee et al., 2004). In contrast, in our study, we observed an upregulation of XIAP and Bcl-xL in both PDAC cells acutely exposed to acidic pH_e_ but a decrease of the cellular levels of FLIP and Mcl-1 following acidic pH_e_ adaptation. These results again display that cell signaling in cancer cells is affected very differently upon short-term and long-term pH_e_ exposure. Diverse dependencies towards pH_i_ acidification to induce apoptosis have been seen in type I and type II cells (Matsuyama and Reed, 2000). In type I cells, apoptosis was overall unaffected upon changed pH_i_ when treated with Fas, while apoptosis was partially suppressed in type II cells when pH_i_ was kept neutral instead of acidic after Fas treatment (Matsuyama and Reed, 2000). This indicates that cytosol acidification may be important to TRAIL-induced apoptosis in PDAC cells, which are known to be type II cells (Hinz et al., 2000; Trauzold et al., 2001). Cytosolic acidification can be blocked by Bcl-2/Bcl-xL (Matsuyama et al., 2000), while we have now shown that acidic extracellular pH lowers the levels of FLIP and Mcl-1, indicating that pH and anti-apoptotic proteins both can regulate each other. Another study showed that overexpressed Bcl-2 in colorectal carcinoma cells was able to lower TRAIL-induced cell death in acute acidic pH_e_ and did not differ highly from pH_e_ 7.4 (Lee et al., 2004). This indicates that cancer cells that already have a high overexpression of anti-apoptotic proteins can escape the TRAIL-sensitizing effect from acidic pH_e_. Panc1 cells show a generally higher expression of anti-apoptotic proteins than Colo357 cells (Trauzold et al., 2003), and this can be partly responsible for the ongoing TRAIL resistance of these cells also in acidic pH_e_. Using orally bioavailable small molecular inhibitors of Bcl-2 family proteins, we have shown that the resistance of Panc1 cells under acidic pH_e_-adapted conditions is highly dependent on Bcl-xL and can be overcome by the treatment with Bcl-xL/Bcl-2 inhibitor Navitoclax. Such effects were much less pronounced in the other pH_e_ conditions. Since the specific Bcl-2 inhibitor Venetoclax did not show the same effects in our current study and also in other studies analyzing TRAIL responses under normal pH_e_ conditions in PDAC cells (Hari et al., 2015; Legler et al., 2018), this confirms that Bcl-xL and not Bcl-2 is responsible for the apoptosis resistance in PDAC cells.

In conclusion, our study has shown that long-term exposition to acidic pH_e_ alone increases TRAIL sensitivity in PDAC, but mainly in already TRAIL-sensitive PDAC cells. The TRAIL-resistant cell line Panc1 decreased both TRAIL-R1 and TRAIL-R2 at the cell surface under long-term acidic pH_e_ conditions, which partly explains their ongoing resistance to TRAIL. Different capacities to quickly adapt and respond to altered pH_e_ in TRAIL-sensitive and TRAIL-resistant PDAC cells could also be observed in the change of TRAIL-induced signaling pathways and the expression of anti-apoptotic proteins. Importantly, acid-adapted Panc1 cells could be sensitized to TRAIL by using an inhibitor of Bcl-xL, again pointing to the decisive role of the mitochondrial amplification loop in these cells. The chosen cell lines, Colo357 and Panc1, have been extensively studied by us and others and are widely accepted models for studying PDAC. Yet, since only two PDAC cell lines were analyzed, the generalization of the conclusions may be limited. Nevertheless, our study gives important insights into the effect of pH_e_ on TRAIL-induced signaling in PDAC cells, improving our understanding of the function of TRAIL receptors in this particularly aggressive cancer.

## Data Availability

The original contributions presented in the study are included in the article/Supplementary Material, further inquiries can be directed to the corresponding author.
