# Supplementary figures and images for: Impact of Extracellular pH on Apoptotic and Non-Apoptotic TRAIL-Induced Signaling in Pancreatic Ductal Adenocarcinoma Cells
Source: Front Cell Dev Biol. 2022 Feb 24;10:768579. doi: 10.3389/fcell.2022.768579 (PMC8907891; doi:10.3389/fcell.2022.768579)

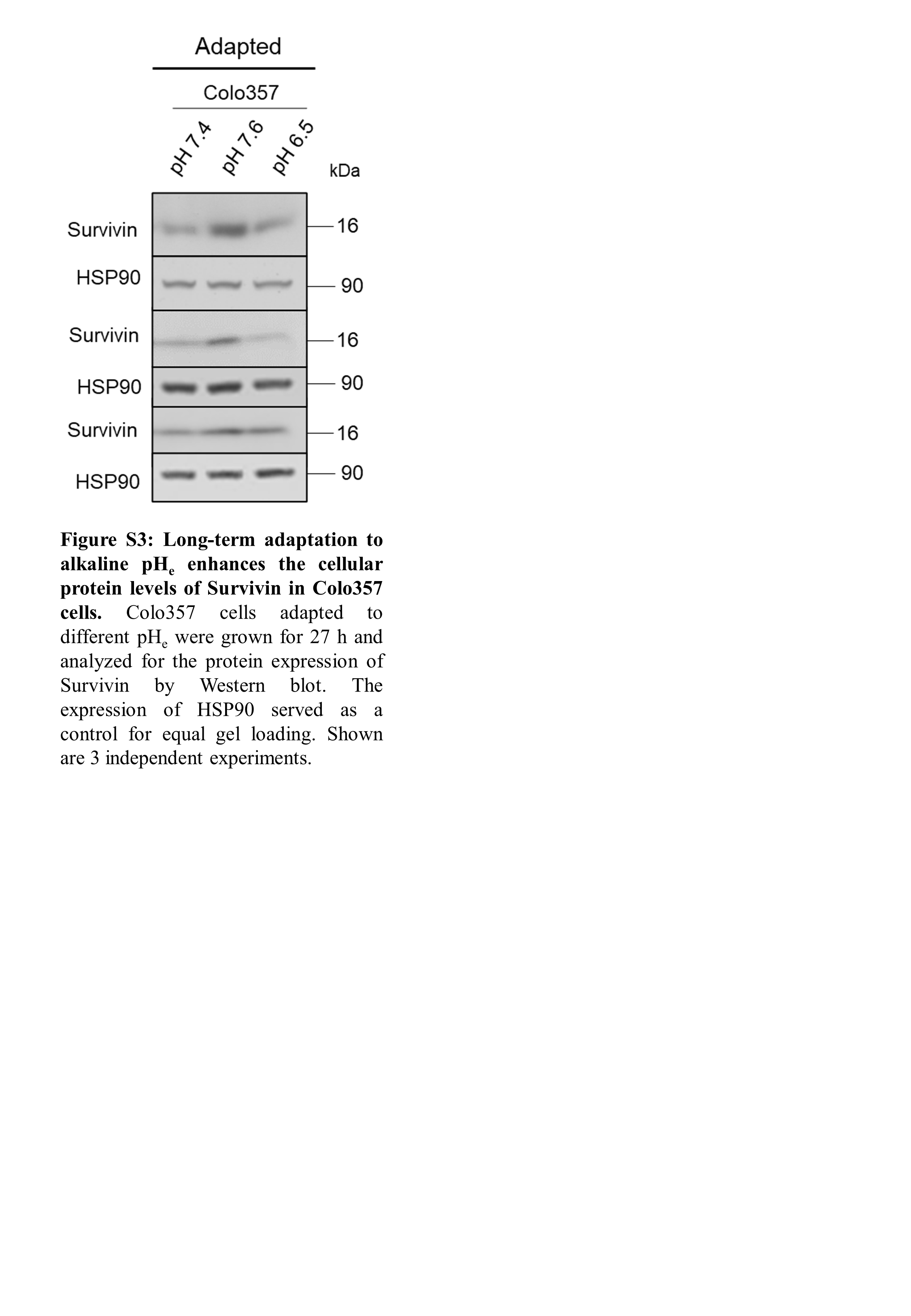

Supplement: Supplementary file 1 [file Image3.TIF]

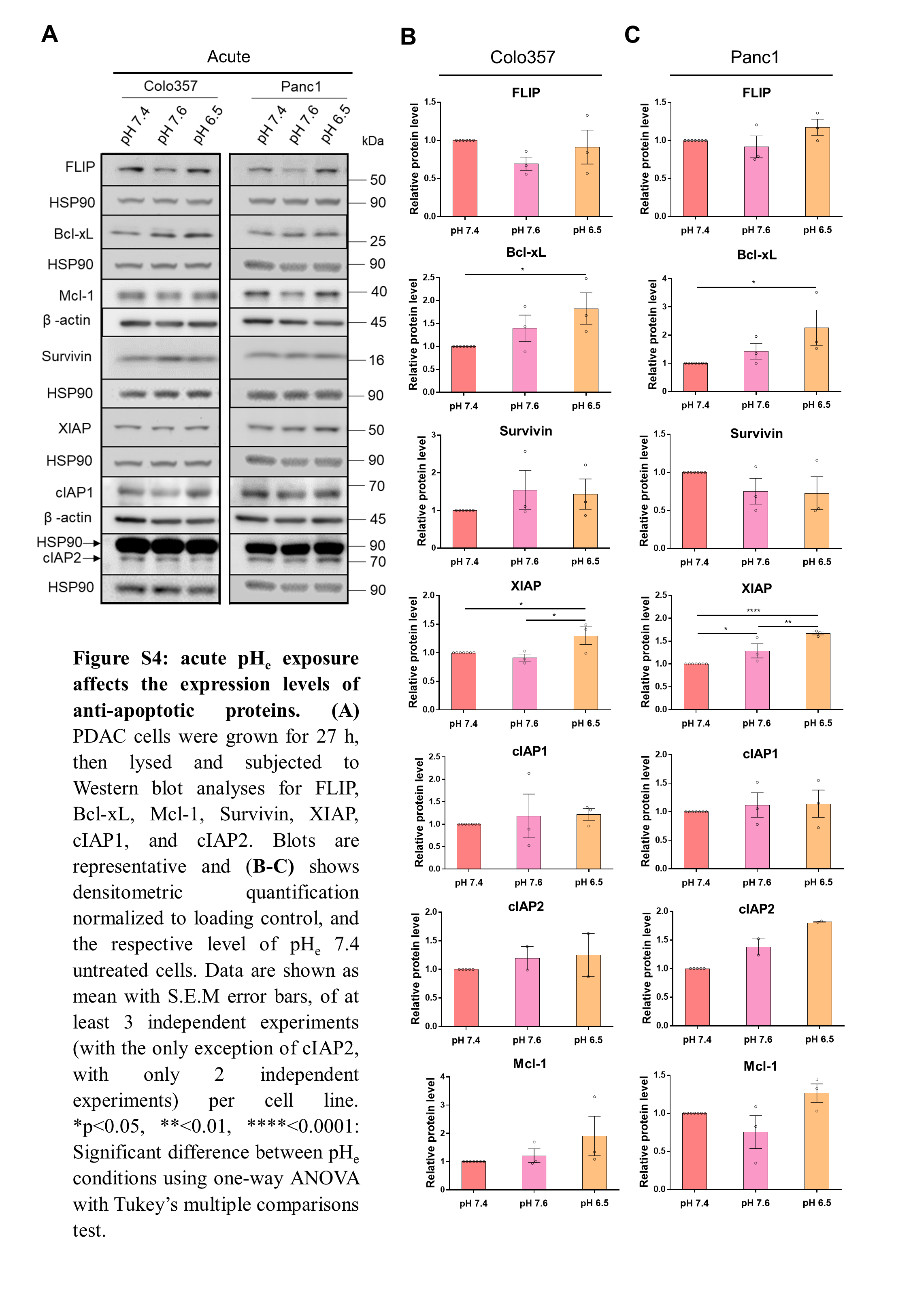

Supplement: Supplementary file 2 [file Image4.TIF]

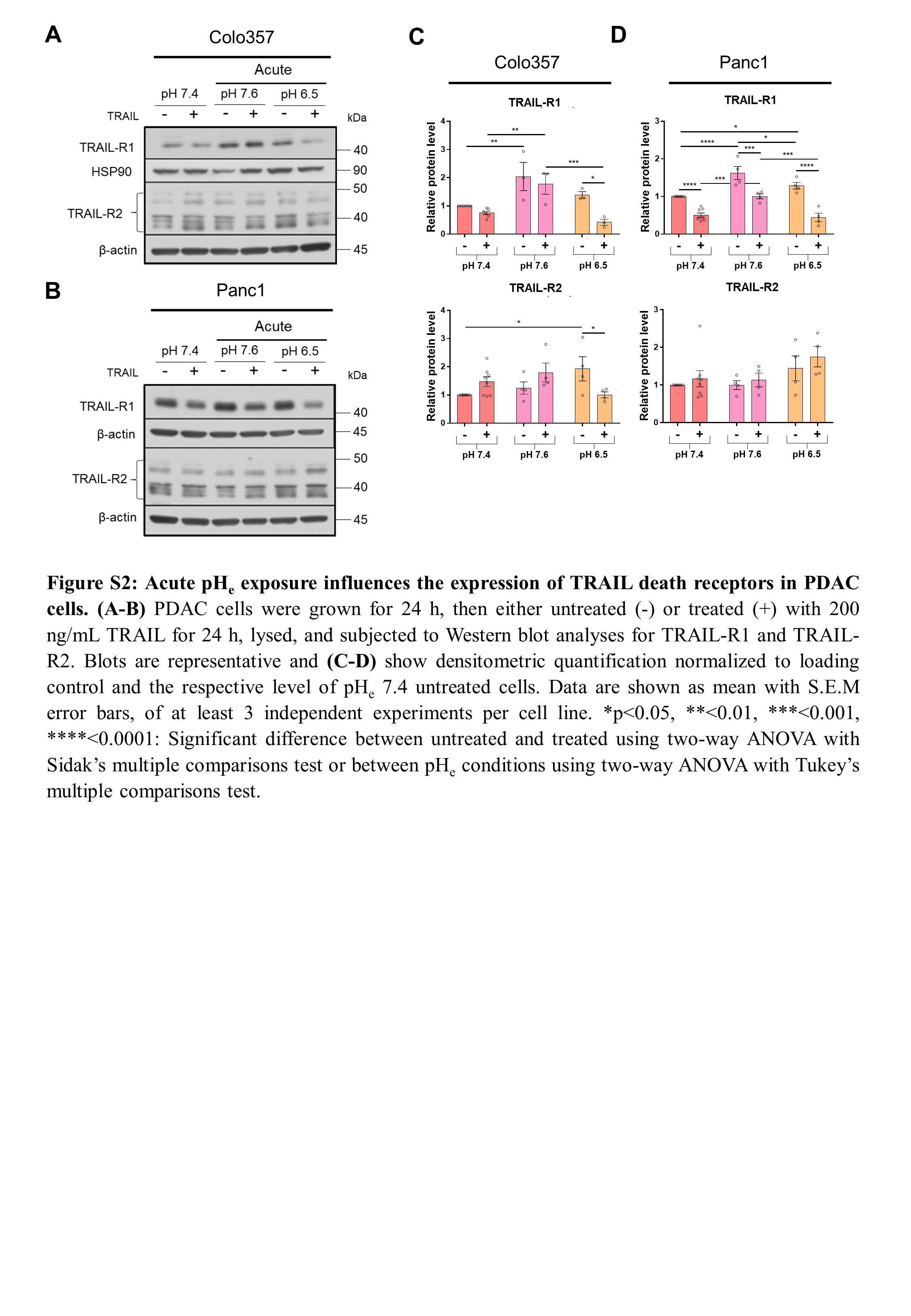

Supplement: Supplementary file 3 [file Image2.TIF]

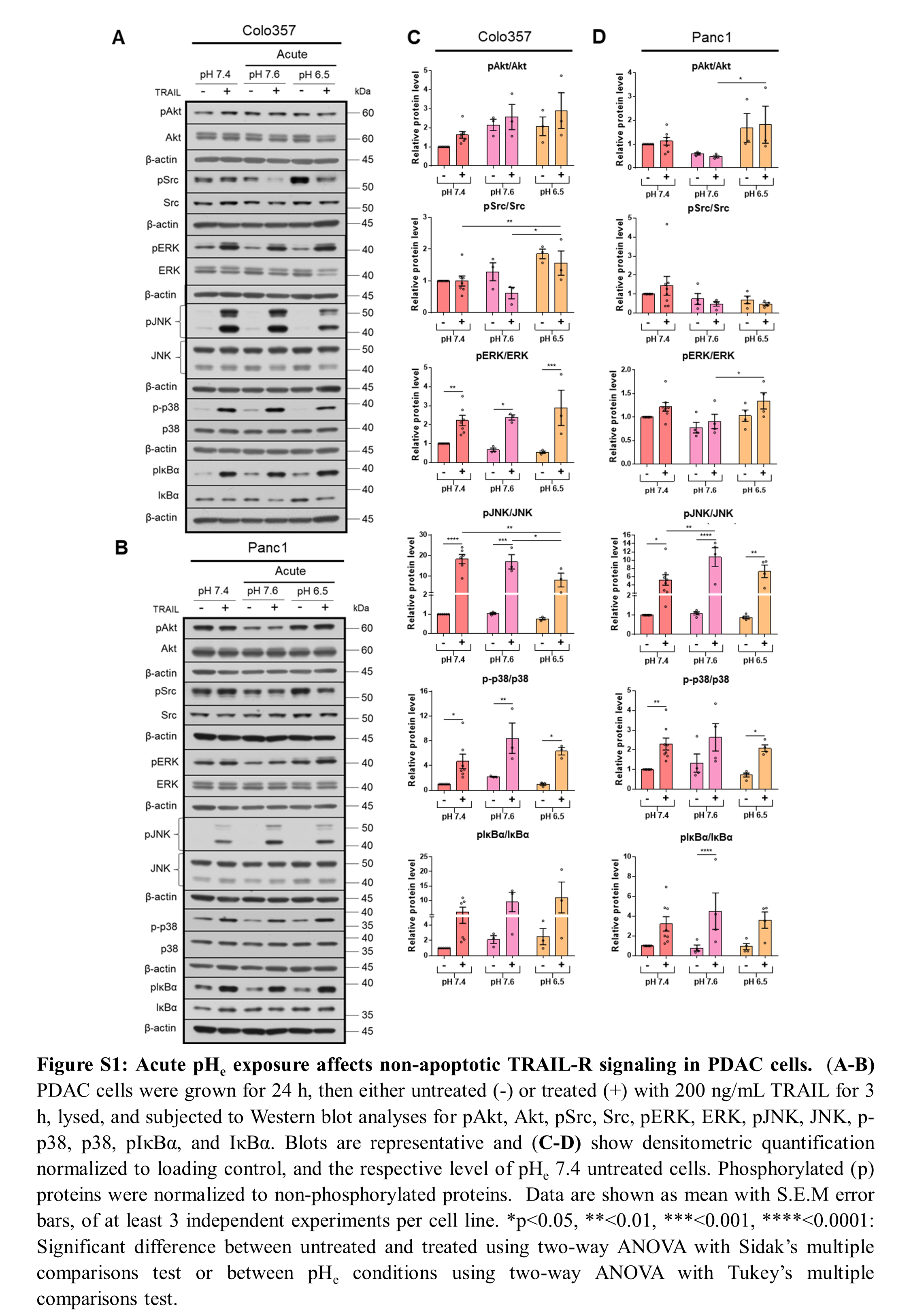

Supplement: Supplementary file 4 [file Image1.TIF]

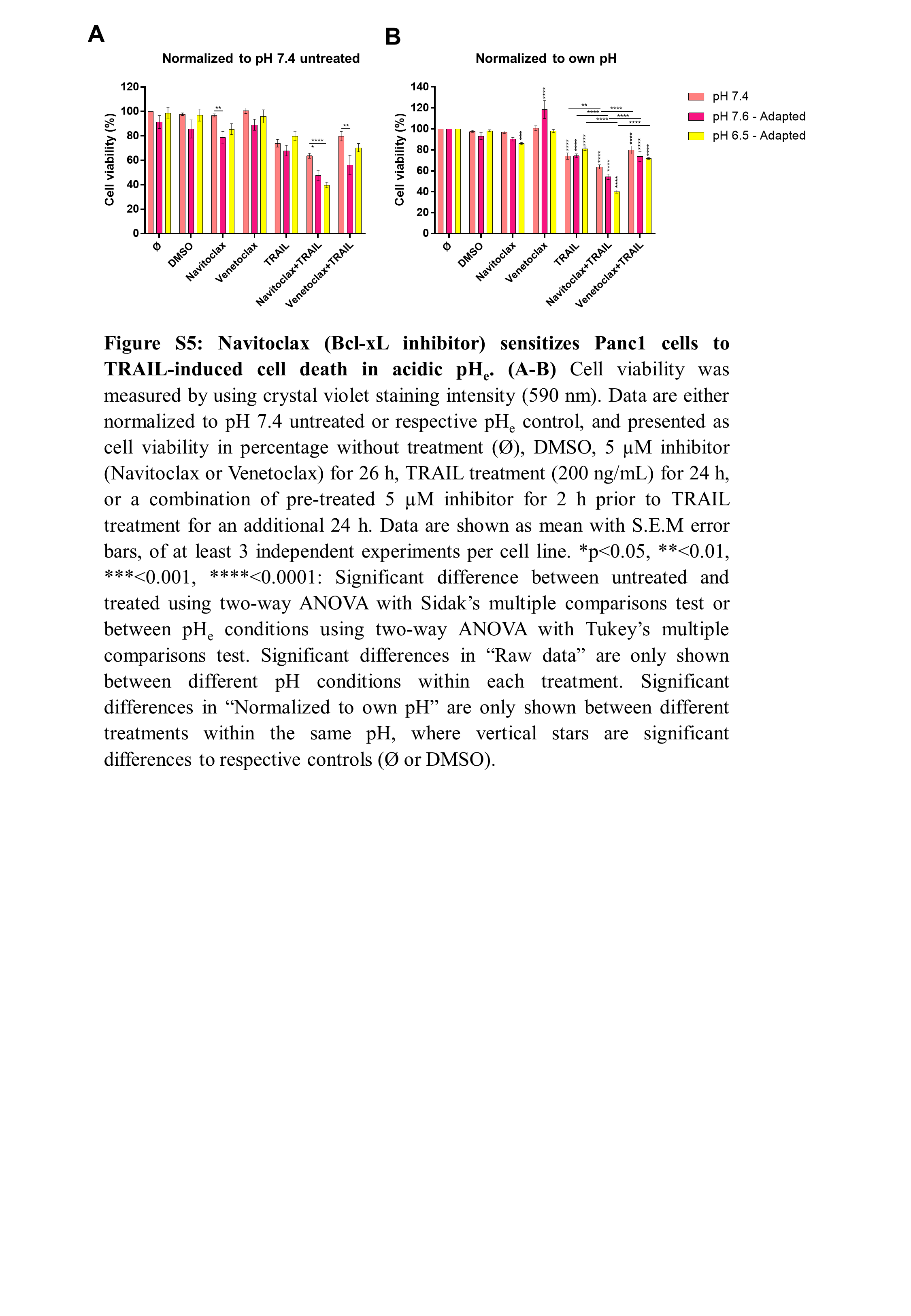

Supplement: Supplementary file 5 [file Image5.TIF]
